# Triggering multiple sclerosis: infection with Epstein-Barr virus activates multiple pre-existing autoreactive B and T cells

**DOI:** 10.1038/s41392-026-02684-7

**Published:** 2026-04-07

**Authors:** Edgar Meinl, Christian Münz

**Affiliations:** 1 Institute of Clinical Neuroimmunology, University Hospital, Ludwig-Maximilians-Universität München, Munich, Germany; 2 Biomedical Center (BMC), Faculty of Medicine, Ludwig-Maximilians-Universität München, Martinsried, Germany; 3 Viral Immunobiology, Institute of Experimental Immunology, University of Zürich, Zurich, Switzerland

**Keywords:** Neuroimmunology, Neurological disorders, Microbiology

Three recent publications^1–3^ show that the Epstein-Barr virus (EBV) activates pre-existing autoreactive T cells by altering the immunopeptidome and inducing molecular mimicry, while also preventing the elimination of autoreactive B cells. These multiple autoimmune responses against the central nervous system (CNS) triggered by EBV might then synergistically precipitate multiple sclerosis (MS) within years after encountering this virus.

Infection with the ubiquitous EBV is strongly associated with the development of MS; in fact, a prior EBV infection is almost a prerequisite for the development of MS. Worldwide, about one of 1000 individuals infected with EBV develops MS, typically 5–10 years after encountering this virus. EBV persists lifelong after infection in about 1 in 10^4^-10^5^ peripheral blood B cells of healthy virus carriers. EBV induces one of the strongest T cellular immune response in humans, causing during 10% of primary infections the immune pathology infectious mononucleosis. However, it is unclear by which mechanism the infection with EBV precipitates MS. Molecular mimicry against different targets and a dysregulated immune response against EBV in the CNS have been proposed.^4^

**Fig. 1:**
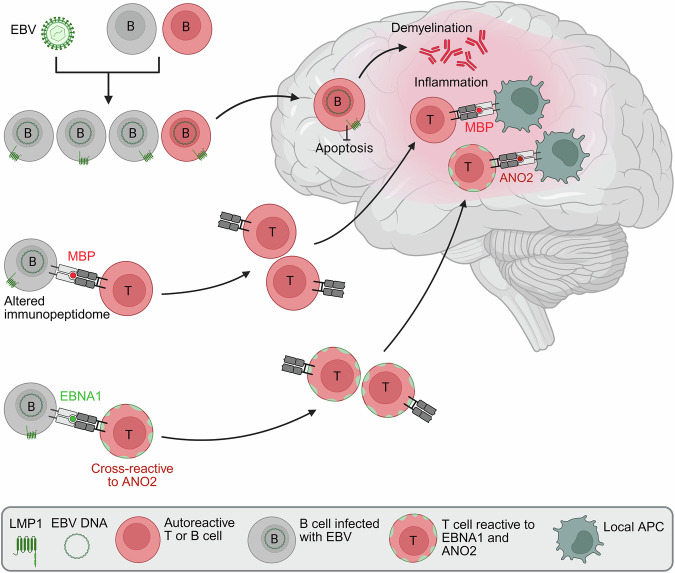
Activation of pre-existing autoimmune cells by EBV jointly triggers MS. This figure illustrates how the findings presented in refs. ^1–3^ synergize to explain the initiation of inflammatory MS lesions. The normal human immune repertoire contains autoreactive T and B cells. EBV-infected B cells acquire the ability to enter the CNS. Upper part: If EBV infects a brain-reactive B cell and this enters the brain, it captures myelin. The EBV-encoded LMP1 protects this B cell from apoptosis, enabling the production of demyelinating antibodies. Middle part: EBV infection of B cells induces the display of an altered immunopeptidome that also includes MBP-peptides. This activates MBP-specific T cells, which divide, and higher levels of MBP-reactive memory B cells were seen in the blood of pwMS. When these MBP-specific T cells enter the brain, they are reactivated by local APCs presenting MBP-peptides, thus inducing inflammation. Lower part: EBV-infected B cells present peptides of the EBV-encoded EBNA-1 and activate T cells to proliferate. Some of these EBNA-1-specific T cells show a cross-reactivity to the autoantigen ANO2. Higher levels of ANO2 reactive T cells were seen in the blood of pwMS. When these T cells that recognize both EBNA1 and ANO2 enter the brain, they get reactivated by local APCs, enhancing the local inflammation. Active MS lesions are characterized by inflammation and demyelination, due to the synergy of autoreactive T cells and demyelinating antibodies. Created in BioRender. Meinl, E. (2026) https://BioRender.com/8ufmdv7

A team around Tobias Derfuss and Nicholas Sanderson combined a novel animal model with the analysis of MS tissue material.^1^ In their animal model, they show that B cells reactive for myelin oligodendrocyte glycoprotein (MOG) enter the brain when there is a local infection and capture myelin. Without further stimulus, these B cells disappear presumably by activation induced cell death that prevents autoimmunity. However, when these B cells transgenically express the EBV-encoded latent membrane protein 1 (LMP1), they overcome this checkpoint, differentiate to antibody (Ab) producing plasma cells and cause local demyelination. LMP1 signals constitutively like CD40 after binding to CD40L on T cells^4^ providing a survival signal for the autoreactive B cells (Fig. 1). In their model, they stimulated the migration of B cells to the CNS by inducing an infection in the brain. Intriguingly, it was recently observed that EBV infected B cells readily migrate to the CNS, suggesting that additional EBV gene products facilitate this homing while LMP1 ensures their survival. In the human part of their study, they found that MOG-reactive B cells are part of the normal immune repertoire. Analyzing MS tissue, they observed LMP1 in B cells in an MS lesion. These intriguing findings link EBV-infection to the development of demyelinating autoantibodies. However, this paper does not tell us what the actual target of the (suspected) demyelinating antibodies in people with MS (pwMS) is. In their animal model, they transferred MOG-specific B cells, and these eventually caused demyelination when they expressed LMP1. In humans, antibodies to MOG constitute a separate disease (MOGAD), which is distinct from MS; in children, MS, but not MOGAD, is linked to EBV infection. The observation by Kim et al.^1^ will encourage further search on the molecular targets of demyelinating antibodies in MS, but also of other autoimmune B cells that might be rescued from cell death by LMP1 in diseases that might also be linked to EBV, such as RA and SLE.^4^

A team around Roland Martin analyzed the peptides presented in the context of MHC class II by EBV-infected B cells from HLA-DR15^+^ pwMS.^2^ The HLA-DR15 haplotype, the strongest genetic risk factor for MS, encodes two non-allelic HLA-DR molecules, called DR2a and DR2b. They determined the peptides presented by DR2a and DR2b, the so-called immunopeptidome. EBV-infection of B cells enhanced the broadness of the immunopeptidome. Within the immunopeptidome of EBV-infected B cells, they found peptides of the CNS autoantigen myelin basic protein (MBP), namely MBP78-90, MBP83-90, and MBP91-106. Both primary and EBV-infected B cells produce a splice variant of MBP, called Golli-MBP, and therefore, B cells present endogenous MBP-peptides on HLA-DR molecules. The identified MBP-peptides in EBV-infected B cells were distinct from the well-known MBP83-99, which has been found previously to be immunodominant for MBP-specific T cells from pwMS and controls.^5^ Analysing the immunopeptidome from MS brain tissue, they found peptides covering basically the complete major myelin isoform of MBP, including three of the peptides derived from EBV-infected B cells. Furthermore, they detected by immunostaining in B cells in follicle-like aggregates in the meninges of pwMS the EBV-encoded proteins LMP1, EBNA2, and gp350. Then they analyzed the T cell response of pwMS and healthy donors to MBP peptides. Since numerous previous studies described MBP-specific T cells in MS and controls at a similar frequency,^5^ they did not compare all T cells, but prepared a fraction of PBMC (CD45RA^-^), which includes monocytes and memory T cells, but not naïve T cells and not B cells. Using these cells, they detected a higher T cell response in the blood of pwMS to the MBP-peptides previously detected in EBV-infected B cells. Furthermore, they expanded CD4^+^ T cells as bulk cultures from the CSF of pwMS that also contained T cells recognizing the MBP-derived peptides derived from EBV-infected B cells. Based on these intriguing findings, they propose the following concept: EBV infection alters the immunopeptidome, leading to the presentation of MBP-peptides by EBV-infected B cells in the context of DR2a and DR2b. This activates preexisting MBP-specific T cells that enter the brain and cause inflammation (Fig. 1).

A team around Tomas Olsson and Olivia Thomas reports cross-reactive T cells recognizing EBNA1 and anoctamin-2 (ANO2) (a calcium chloride channel) in humans and mice.^3^ This extends their previous observation of an antibody-cross-reactivity between ANO2 and EBNA1 that was associated with the risk of developing MS. The ANO2 sequence of T cell cross-reactivity lies outside the one for antibody cross-reactivity at 140–150. To analyze the T cell response, they have used an antigen-coupled bead assay, which is very sensitive and has the advantage that the applied antigen is naturally processed before activating the T cells. While they detected ANO2-specific T cells also in about 15% of controls, they report them in more than half of pwMS (57%). In an animal model, they could show that ANO2-specific T cells enhance encephalitis, which was mediated by an autoimmune response to another CNS-expressed protein, proteolipid protein. They develop the concept that EBV triggers an immune response against EBNA1, which is partially cross-reactive to ANO2, and this permeabilizes the blood-brain barrier to increase CNS inflammation, maybe in synergy with other autoreactivities (Fig. 1). In support of this concept, an enhanced T cell response to EBNA1 in pwMS has previously been observed.

How abundant is EBV in MS lesions, and is the presence of EBV in lesions required to drive MS? After EBV was reported in large proportions of B cells in MS lesions, this was discussed controversially by neuropathologists. Recently, different studies reported EBV proteins in MS lesions using immunostaining, including.^1,2^ So the picture is emerging that EBV may not be continuously present in inflammatory MS lesions, but EBV gene products can be found at least at some stages in some MS lesions in the minority of B cells, but enriched compared to the low frequencies in peripheral blood. In fact, none of the three concepts presented^1–3^ postulates and requires the continuous presence of EBV in MS lesions (Fig. 1).

Which autoimmune responses are relevant in MS? Multiple autoantigens for MS have been studied over decades.^5^ MBP (the focus of ref. ^2^) is a prominent candidate, because it is highly encephalitogenic in many species. ANO2 is added to autoimmune targets in MS by showing that both an antibody and a T cell response to ANO2 are enhanced in pwMS^3^ and ANO2-specific T cells enhanced autoimmune encephalitis induced by another CNS-derived protein. Overall, there is strong evidence that the inflammatory and demyelinating MS lesions are caused by a synergy of T and B cells, which may recognize different targets. In different animal models, T cells cause inflammation, but no demyelination, while antibodies alone are not pathogenic. However, when demyelinating Abs and encephalitogenic T cells, which may recognize different autoimmune targets, were combined, then inflammatory and demyelinating lesions were observed as in MS.^5^ The three papers presented here nicely synergize to support this concept of multiple hits by autoreactive T and B cells during MS development (Fig. 1). One hit is by autoreactive T cells, driven by altered antigen processing^2^ and/or molecular mimicry^3^ after EBV-infection. A second hit is mediated by the Abs derived from autoreactive B cells that escape activation induced cell death due to EBV infection^1^ or by Abs that show a cross-reactivity between an EBV-encoded protein (most evidence is for EBNA1) and an antigen expressed in the CNS.^4^ It is tempting to speculate that multiple such EBV-induced events have to take place in the same patient to induce MS, explaining the fortunately rare development of MS after EBV infection. Remarkably, while the MS-related inflammatory CNS-diseases MOGAD and NMOSD are diagnosed based on the presence of one specific autoantibody for each disease, no test for autoimmunity (neither at the T cell nor at the B cell level) is currently useful for the diagnosis of MS. The reason for this might be that classical MS is driven by the synergy of multiple – EBV triggered – autoreactive B and T cells, recognizing CNS antigens that might differ between individual pwMS (Fig. 1).

Together, these three papers^1–3^ show that the Epstein-Barr virus (EBV) activates by distinct mechanisms multiple autoimmune responses, which might synergistically trigger MS.
